# Socioeconomic inequities and cardiovascular disease-related disability in China: A population-based study

**DOI:** 10.1097/MD.0000000000004409

**Published:** 2016-08-12

**Authors:** Zhenjie Wang, Chengfu Cao, Chao Guo, Gong Chen, Hong Chen, Xiaoying Zheng

**Affiliations:** a Institute of Population Research/WHO Collaborating Center on Reproductive Health and Population Science, Peking University, Haidian District; b Department of Cardiology, Peking University People's Hospital, Beijing, People's Republic of China.

**Keywords:** cardiovascular disease, China, disability

## Abstract

The prevalence of disability has changed along with aggressive economic development in China. However, socioeconomic inequalities associated with cardiovascular disease (CVD)-related disability have not been explored. This is the first study to explore CVD-related disability among persons aged 45 years and older in China.

Data were taken from the 2006 Second China National Sample Survey on Disability, which was a nationally representative, population-based survey. To derive a nationally representative sample, the survey used multistage, stratified, and cluster random sampling with probability proportional to size. We used standard weighting procedures to construct sample weights that considered the multistage, stratified, and cluster sampling survey scheme. Associations between CVD-related disability risk and socioeconomic inequality were examined using logistic regression.

In this study, the weighted prevalence of CVD-related disability was 1.84 per 100 persons (95% confidence interval [CI]: 1.80–1.89), and 73% of CVD-related disability consisted of a single disability, including speech, physical, and intellectual disabilities, whereas 23% of CVD-related disability consisted of multiple disabilities, that is, any combination of speech, physical, and intellectual disabilities. A higher risk of CVD-related disability was observed among rural residents than urban residents as well as among males than females. Age presented consistent increased associations with CVD-related disability. Education inequality was strongly associated with the risk of multiple disabilities.

To address the challenge of CVD-related disability in China, the government should adjust its strategies for health care systems to prevent disability. The widening discrepancy between urban and rural areas indicates that the most important priorities for disability prevention in China are to reinforce health promotion in the working age population and to improve health services in rural communities.

## 1 Introduction

Cardiovascular disease (CVD) is the most common cause of death worldwide and is also a major contributor to the growing public health epidemic of chronic disease. ^[1]^ Much of the disease and disability burdens from chronic diseases are found in the population younger than 70 years old. ^[2]^ A major outcome of CVD disability is a common health issue in both developed and developing countries. ^[3]^ The World Health Organization (WHO) estimates that approximately 650 million people live with disabilities. ^[3]^ In China, approximately 85 million people live with disabilities or another condition that affects their daily lives and social activities. ^[4]^ It is estimated that the number of disabled people increased from 52.7 to 84.6 million, and the weighted prevalence of disabilities increased from 4.9% to 6.5% in 1987 to 2006, accompanying Chinese development. ^[5]^

Several epidemiological studies have been conducted to specifically analyze different types of disorders and disabilities. [^6^ ^7^ ^8^ ^9^ ^10^ ^11^ ^12^ ^13^ ^14^ ^15^] Mental disorders are an example of this. A recent study suggested that the prevalence of mental disorders has increased rapidly in China. ^[15]^ Wealth inequality is related to disability risk, especially for those with a mental disability. ^[16]^ In China, the estimated number of CVD cases was 270 million in 2014, and CVD is the most common cause of death in China. ^[17]^ Although China is challenged by CVD, the existing studies on CVD [^17^ ^18^ ^19^ ^20^] provide no scientific evidence of socioeconomic inequities in CVD-related disability risk in China. Disability is a common serious health outcome that affects daily life and social participation. In the present study, we used a nationally representative survey on disability to explore the prevalence of CVD-related disability and the socioeconomic inequities in CVD-related disability risk in China. ^[21]^

## 2 Methods

### 2.1 Data source

In the present study, we used the latest data on disability from the nationally representative, population-based 2006 China National Sample Survey on Disability. The survey used multistage, stratified, and random cluster sampling in which the probability was proportional to size to derive a nationally representative sample. Within each province, sampling strata were defined based on subordinate administrative areas, local geographical characteristics, or local gross domestic product, where appropriate, to allow for anticipated regional variability. The survey protocol and questions were reviewed by leading national and international experts, and the sampling scheme was reviewed by experts from the Division of Statistics of the United Nations. The survey covered all provincial administrative areas in Mainland China, excluding Hong Kong, Macau, and Chinese Taipei. The final sampling ratio was 1.93 per 1000 of the total population. ^[21]^ Details of the design are described elsewhere. ^[5]^

### 2.2 Ethics

The survey was approved by the State Council, conducted in all of the province-level administrative regions of mainland China, and performed by the Leading Group of the China National Sample Survey on Disability and the National Bureau of Statistics. The survey was conducted within the legal framework governed by statistical law in China. All of the respondents provided consent to participate in the survey.

### 2.3 Interviewing procedures and data quality

A pilot study was conducted in different provinces before each survey. Strict quality control measures were implemented at every step of the survey, from the drafting of the sampling frame to field sampling, from the filling out of the questionnaires to the checking of the returned forms, and from data input to the checking of data quality. ^[21]^ During data collection, trained field interviewers, including a team leader, a deputy team leader, a deputy medical team leader, investigators, statisticians, and medical doctors with expertise in otolaryngology, ophthalmology, surgery/orthopedics, psychiatry, and pediatrics, used a structured questionnaire to inquire about disabilities. Those who responded “yes” to any of the questions were assigned to the designated physician for further disability screening and confirmation. The designated physician performed a medical examination, followed diagnostic manuals to make a final diagnosis, if any, and confirmed the primary causes. Respondents with multiple positive diagnoses were examined by multiple specialists (a separate physician for each disability).

After the field investigations concluded, the teams made home visits to conduct surveys in the quarters chosen for postsurvey quality checks to calculate the overall errors in the survey. The results of the quality checks showed that the omission rate of the resident population was 1.31 per 1000 persons; the omission rate of the disabled population was 1.12 per 1000 persons. ^[21]^

### 2.4 The definition of CVD-related disability

Disabilities were defined and classified by the expert committee of the Second China National Sample Survey on Disability, based on the WHO International Classification of Functioning, Disability and Health. ^[22]^ The definition of CVD-related disabilities used in this study is presented in Table 1. Disabilities were diagnosed by medical doctors who used a structured questionnaire to inquire about disabilities according to the WHO International Classification of Functioning, Disability and Health. ^[22]^ The causes of CVD-related disability were as follows: cerebral infarction, cerebral hemorrhage, cerebrovascular disease, and peripheral vascular disease. ^[21]^ Multiple CVD-related disabilities were defined as more than 1 type of disability, as presented in Table 1.

**Table 1:**
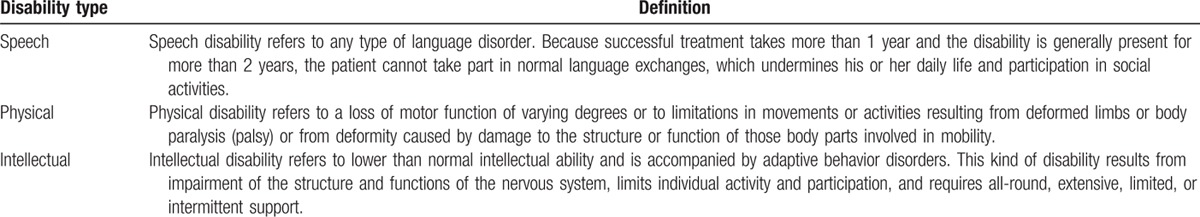
Study definitions for different disability types. ^[5]^.

### 2.5 Study variable definition

We defined the status of CVD-related disabilities as binary (i.e., yes or no); the status of a single CVD-related disability as binary (i.e., yes or no); the status of multiple CVD-related disabilities as binary (i.e., yes or no); age group as 45 to 49, 50 to 54, 55 to 59, 60 to 64, 65 to 69, 70 to 74, 75 to 79, or 80+; gender as male or female; residential area as urban or rural, according to where the subjects were living; ethnicity as Han or other; education level as never attended school, primary school, or junior high school and above; marital status as never married, divorced/widowed, or married; household size as 1 to 3, 4 to 6, or 7 to 9 (persons/household); living arrangement as living with others or living alone; and current employment status as binary (i.e., employed or unemployed).

### 2.6 Data analysis

For the final analysis, we selected a population aged 45 and older because the prevalence of CVD-caused deaths is greatly increased in this age range in China. ^[23]^ We used standard weighting procedures, calculating the inverse probability of inclusion for an individual survey respondent in the multistage sampling frame to construct sample weights taking into account the complex survey sample design. ^[24]^ Descriptive statistics were used to present the sample characteristics and population numbers weighted by various socioeconomic characteristics. A logistic regression model was used to calculate the adjusted odd ratios (ORs) and 95% confidence intervals (CIs) of each socioeconomic characteristic. As multicollinearity might exist in the models, we also used stepwise logistic regression to estimate the ORs and 95%CIs. Statistical significance was set to a 2-sided *P*-value <0.05. The statistical analyses were conducted with SAS v. 9.2 (SAS Institute, Inc., Cary, NC). The procedures SURVEYFREQ and SURVEYLOGISTIC were used to perform the data analyses. The stepwise method in the procedure SURVEYLOGISTIC was implemented by referencing the SAS Model Selection Macros for Complex Survey Data Using Proc Surveylogistic/Surveyreg. ^[25]^

## 3 Results

### 3.1 Characteristics of the subjects

The selected characteristics of the populations with and without CVD-related disabilities are presented in Table 2. The weighted prevalences of CVD-related disability, a single CVD-related disability, and multiple CVD-related disabilities were 1.84 per 100 persons (95% CI: 1.80–1.89), 1.29 per 100 persons (95% CI: 1.25–1.32), and 0.57 per 100 persons (95% CI: 0.55–0.60), respectively. In the current study, females, rural residents, people living with others, and persons of Han nationality accounted for the majority of the healthy population. Male subjects, rural residents, disabled people with low education level, and the unemployed accounted for the majority of the single CVD-related disability and multiple CVD-related disabilities populations.

**Table 2:**
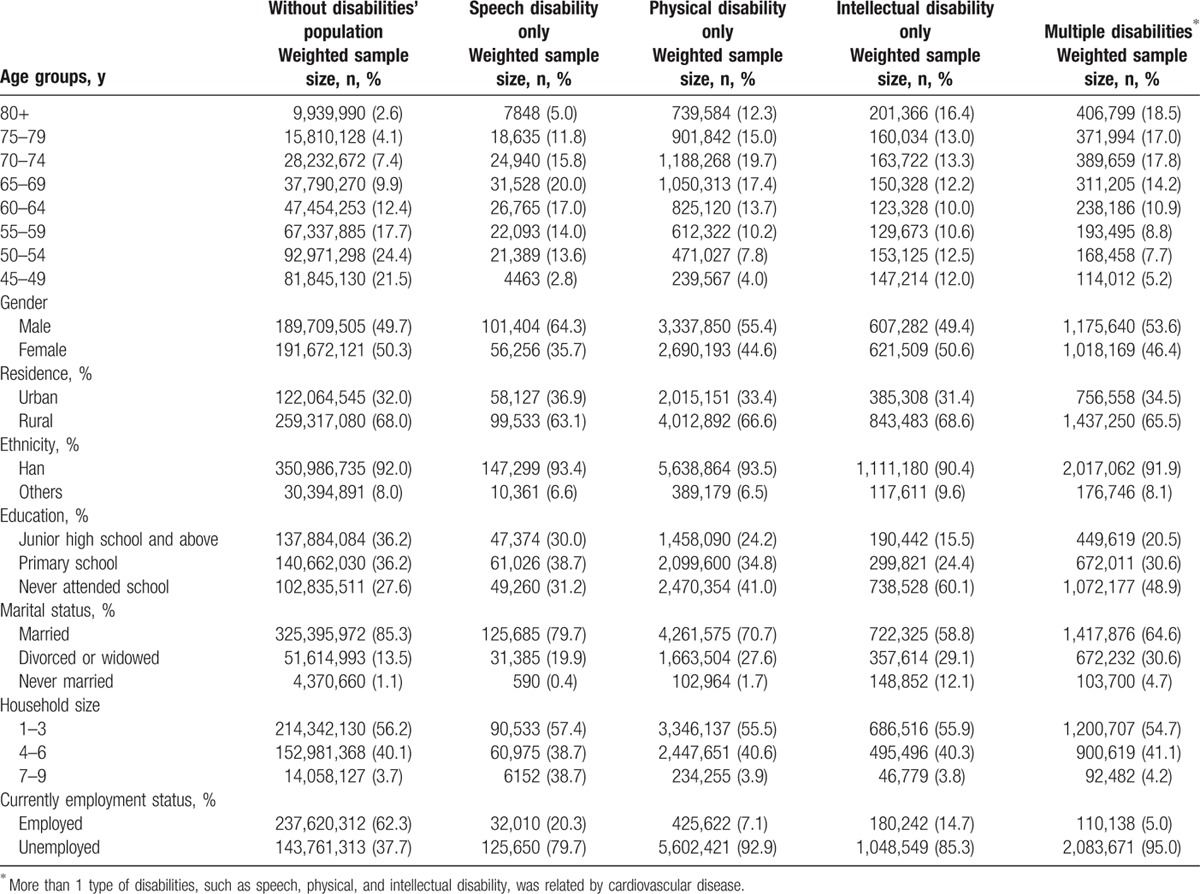
Characteristics of weighted healthy population and cardiovascular disease-related disability population.

### 3.2 Associations between socioeconomic factors and CVD-related disability

There was a strong and consistent association of socioeconomic factors, such as age group, gender, residence area, and education, with CVD-related disability, including single and multiple CVD-related disabilities (Table 3). Compared with people aged older than 80 years, the probability of having a CVD-related disability gradually decreased from 24% (*P* < 0.05) in those aged 75 to 79 to 64% (*P* < 0.05) in those aged 45 to 49. Male subjects were more vulnerable than female subjects. Subjects who lived in rural areas (OR [95%]: 1.46 [1.37–1.55]) or who had less access to education had a higher risk of CVD-related disability than those who lived in urban areas or had high education levels, respectively. Similar associations were observed between a single CVD-related disability and multiple CVD-related disabilities.

**Table 3:**
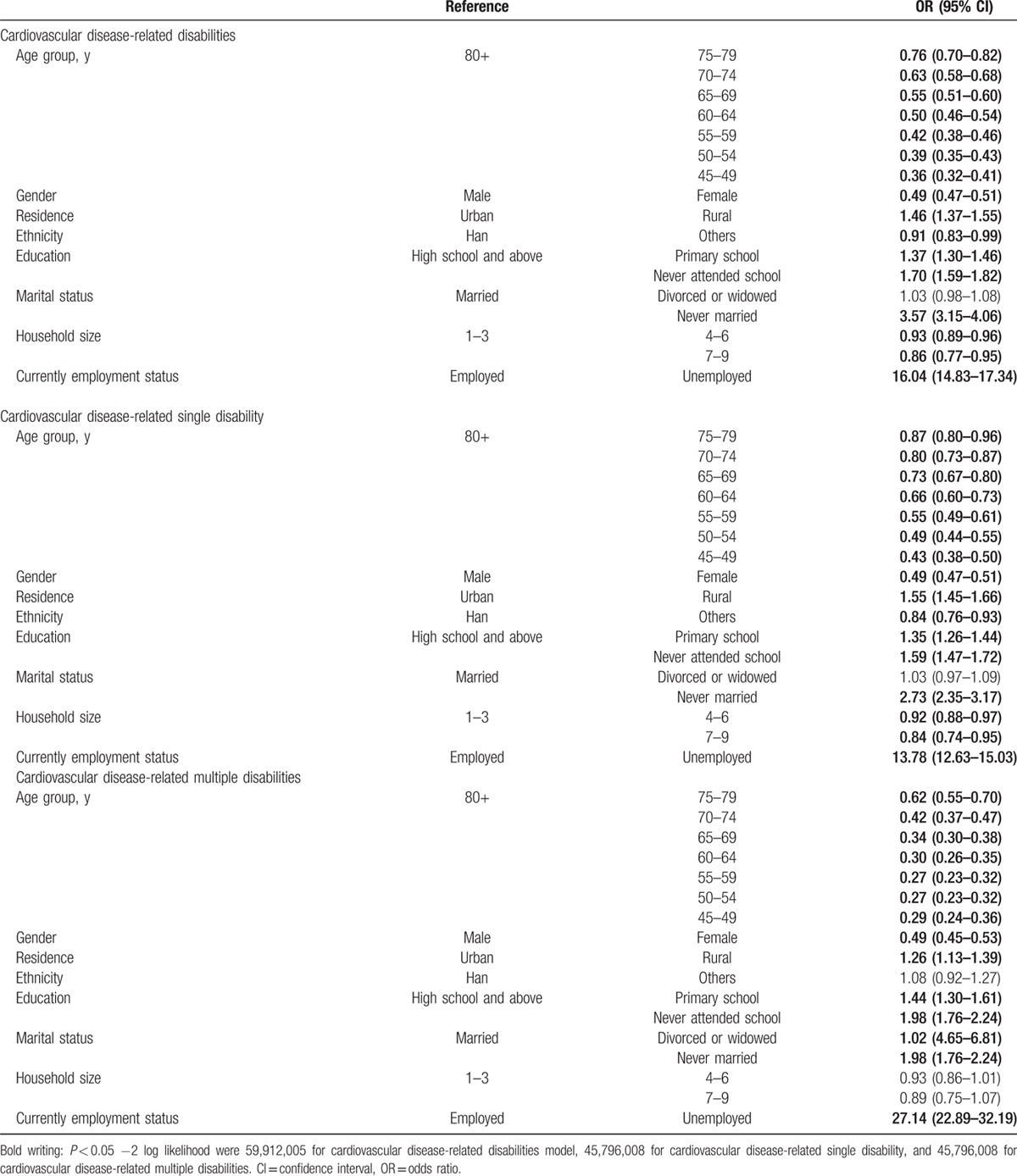
Associations of cardiovascular diseases-related disabilities in Chinese population.

We repeated the analysis of the associations between socioeconomic factors and psychiatric disability using stepwise logistic regression. In addition to the subcategory of household size (4–6 persons per household), other socioeconomic factors of CVD-related disabilities, including a single and multiple CVD-related disabilities, were included in the final models.

## 4 Discussion

### 4.1 Main findings and their significance

The role of socioeconomic inequities among those with and without a CVD-related disability has rarely been investigated in China. We used detailed personal interviews and professional examinations of disabilities from a 2006 nationally representative sample. We obtained valuable and unique data on CVD-related disability among the Chinese population. The weighted prevalence of CVD-related disability was 1.84 per 100 persons. Furthermore, we observed strong associations between socioeconomic factors (e.g., age group, gender, residence area, and education level) and CVD-related disability.

### 4.2 Comparison with other studies and implications of the findings

The prevalence of CVD-related disability, including different types of single disability and multiple disabilities, was 1.84 per 100 persons (95% CI: 1.80–1.89). The prevalence of CVD-related death was greater than 0.3 per 100 persons in upper-middle-income countries. ^[1]^ According to our results and those of other epidemiology studies, CVD-related disability is a serious public health issue in China because the disabled population is a huge economic burden. For example, mental disability amounts to a country-wide gross domestic product (GDP) loss amounting to CNY 2681 billion in China. ^[26]^ Most of the disabled people developed another CVD-related disability within a very short amount of time in the current study, which suggests that China faces not only the huge socioeconomic burden of disability but also the under-development of the health service system. For example, the number of health professionals increased from 3,608,618 in 1987 to 4,728,350 in 2006, ^[27]^ and during the same time period, the Chinese population increased by 20%. ^[26]^ In 2006, 44.8% of the urban population and 79.1% of the rural population did not have any health care insurance. ^[28]^ The development of health services did not keep up with the population increase. The disabilities social security system and the rehabilitation system in China might subsequently result in the development of multiple disabilities. The majority of people with disabilities never access rehabilitation services and health care, ^[29]^ which might result in the advancement of these individuals to multiple disabilities. Moreover, preventive disabilities services are undeveloped. Therefore, the health services system must be improved, especially for those with CVD-related disabilities. The prevalence of CVD-related death almost doubles every 5 years among people aged 45 and older in China. ^[23]^ In this study, the prevalence of CVD-related disability increased across the subjects’ age groups. The trend of prevalence gradually increased in contrast to the trend of CVD-related death. One potential reason is that the awareness of disabilities in China has increased.

Socioeconomic inequalities in health were observed among various socioeconomic statuses in recent decades, [^30^ ^31^ ^32^] including race, ^[33]^ gender, [^34^ ^35^ ^36^] education,[ ^37^ ^38^] occupation, and household income,[ ^39^ ^40^] but some of these inequalities are modifiable. The incidence of mobility disability was clearly reduced among those with a higher level of education compared with those with a lower level of education. ^[37]^ This similar association was also observed across European countries. ^[37]^ Moreover, socioeconomic inequalities were reduced by improving educational opportunities, income distribution, health-related behavior, and access to health care. ^[41]^ Additionally, wealth inequalities appeared to be more strongly associated with health issues, that is, cardiovascular and all-cause mortality, than other socioeconomic position factors.[ ^31^ ^32^] An epidemiological study suggested that higher education provided protection against developing a disability but provided less benefit when a disability was already present, especially for older people. ^[42]^ The study also found a high incidence of disability among those who attained a lower educational level (i.e., less than secondary education) in the population. Other studies have suggested a gradient in disability by education, occupation, and material living standards.[ ^43^ ^44^] In the present study, we observed results consistent with previous epidemiological studies when the surveys were analyzed separately and together with CVD-related disability.

Urban-rural differences are more salient in developing countries than in developed countries. The limited studies that are available examined the relationships between urban/rural residence and disability in developing countries, specifically CVD-related disability. ^[45]^ In our study, one striking finding was the urban–rural discrepancy in CVD-related disability, including single CVD-related disability and multiple CVD-related disabilities. Potential explanations for the widening urban–rural gap in disability risk and prevalence in China include: that rural residents with multiple disabilities are unable to migrate to urban areas; that in rural areas, the lifestyle is harder, the working standards are poorer, and the availability of healthcare and rehabilitation services are limited; ^[46]^ and that a large majority of the poor population are nonsalaried or unemployed, and a significant proportion are at risk of impoverishing health expenditures. ^[47]^ The discrepancy observed in the present study was consistent with the results from the Fourth National Health Services Survey in 2008, which found higher rates of disabled people among rural residents. ^[48]^ Although the health services system is gradually developing, it still has not caught up with residents’ health care demands and utilization, especially for those with disabilities living in rural areas. Moreover, several lifestyle factors are associated with income, social class, education, or deprivation, and these factors cluster together in a population; many of these factors are the “causes of the causes” and should be the focus of stronger policy action on diseases.[ ^49^ ^50^] Our results suggest that the government should adjust and improve the health services system, create supportive environments, and change the patterns of Chinese life, according to local needs, social culture, and economic systems, [^51^ ^52^ ^53^] especially for those living in rural areas. Additionally, previous studies suggested that sedentary occupations were related to CVD. Sedentary occupations appeared to be linked to obesity in the middle-aged population. ^[54]^ Occupation was not considered in the present study. The proportion of individuals who had a sedentary occupation was 5.3% of the population without a disability and 0.001% of the population with a disability. According to the WHO Study on global AGEing and adult health, women were more disadvantaged, showing a higher prevalence of disability across all age groups. ^[35]^ However, men had a higher risk of CVD-related disability than women in the current study. In China, as observed in the present study, CVD and mortality from CVD are much less prevalent in women. ^[17]^

### 4.3 Strengths and limitations

This study provided a broad understanding of CVD-related disability and its relationship with different key socioeconomic statuses in China. The current study used a large, representative, population-based sample that covered all of the provincial areas. In addition, every subject of the selected households was interviewed by interviewers face-to-face. Subjects who agreed to participate in the study underwent a screening for disabilities by the interviewers, and those suspected of having a disability were then examined and diagnosed by doctors. However, the present study also had some weaknesses. The more stringent definition of disability used in the survey resulted in a low prevalence of CVD-related disability, which should serve to inform future studies. The disability survey did not cover all CVD causes, which also contributed to the underestimation of CVD-related disability. In the current study, CVD diseases that could not be identified might cause bias, which should be considered in further studies. Standardized quality control schemes were in place during the field implementation, such as training of the interviewers and crosschecking of the returned survey responses by contacting survey participants, resulting in little response bias. Additionally, the cross-sectional design does not provide direct evidence of causality.

## 5 Conclusions

Our results are beneficial for developing a better understanding of CVD-related disability and socioeconomic indicators in China. The widening gap in the prevalence rates of CVD-related disability between rural and urban areas may have important practical implications for China. In addition, the study results may help the government to adjust strategies aimed at assisting individuals and communities and improving health care systems in the future.
